# Youth perspectives on information and trust during COVID-19: evidence from Egypt

**DOI:** 10.3389/fdgth.2025.1563203

**Published:** 2025-09-08

**Authors:** Salma Seddik, Ahmed Taha Aboushady, Ahmed Nawwar, Omar Emam, Mahmoud Hemida, Mennatallah Zohny, Adham Ramadan, Eslam Aboismail, Noha M. AbuBakr Elsaid, Amira Hegazy

**Affiliations:** ^1^ Kasr Al Ainy Hospital, Faculty of Medicine, Cairo University, Cairo, Egypt; ^2^ Faculty of Medicine, Cairo University, Cairo, Egypt; ^3^ Faculty of Medicine, Alexandria University, Alexandria, Egypt; ^4^ Department of Computer Science and Engineering, University of Louisville, Louisville, CO, United States; ^5^ Department of Public Health and Community Medicine, Faculty of Medicine, Cairo University, Cairo, Egypt; ^6^ Proton Therapy Center, MD Anderson Cancer Center, University of Texas, Houston, TX, United States; ^7^ Department of Public Health and Community Medicine, Faculty of Medicine, Suez Canal University, Ismaïlia, Egypt

**Keywords:** COVID-19, policy, trust, infodemics, youth, Egypt

## Abstract

**Introduction:**

The detrimental effects of the COVID-19 global pandemic have significantly changed the world's view on enacting policies. Egypt has adopted various protocols and measures to control the spread of its causative virus, SARS-CoV-2. This study aims to examine the public trust in decision-makers and help address possible gaps between the sources of information, theoretical guidelines, implementation, and the confidence of youth in response to the pandemic by the main actors involved.

**Methods:**

An online structured cross-sectional survey was conducted among Egyptian youth during the early COVID-19 pandemic. The questionnaire collected variables, including socio-demographic characteristics, sources of information, and the respondent's confidence in these sources.

**Results:**

Out of 406 online respondents, males and females represented, 41.8% and 58.6%, respectively. Ninety-five percent of the respondents received a university or postgraduate education, of which 63.5% were in the medical field, and 82.3% lived in rural areas. Over forty percent reported their source of information was T.V., and 30.3% relied on social media, despite most of the respondents being unconfident in both. In addition, 12.8% and 5.9% reported that their source of information was the Ministry of Health and the WHO, respectively. Over seventy-seven percent of the respondents were concerned with the decisions taken by key actors in the pandemic situation, while 15% were neutral, and 5.4% were not concerned.

**Conclusion:**

Infodemics is a substantial public health threat. Public health authorities and governments should take action to ensure comprehensive health information literacy and develop information technology strategies that promote access to evidence-based information.

## Introduction

COVID-19, caused by the novel coronavirus SARS-CoV-2, has significantly impacted Egypt. The first case of COVID-19 in Egypt was reported on February 14, 2020, and since then, the number of cases and deaths has steadily increased (World Health Organization). As of February 19, 2023, the World Health Organization (WHO) in Egypt has reported 634,631 confirmed cases and 35,661 deaths (1). A vaccination campaign was then initiated and implemented to mitigate the spread of COVID-19. As of February 19, 2023, Egypt had administered a total of 41,652,868 doses of the COVID-19 vaccine (2).

The COVID-19 pandemic had a significant impact on Egypt. The Egyptian authorities have implemented various measures to control the spread of the virus, including restrictions on public gatherings, curfews, and mandatory masking in public spaces (3, 4). Particularly the Egyptian health system and economy, by overwhelming the health system and health providers and halting the tourism industry, which has been one of the country's key sources of revenue (3, 4). In addition, the government has implemented various measures to support businesses and individuals affected by the pandemic, including financial support to enterprises (5).

The pandemic and its associated measures have affected people of all ages, including youth. The pandemic has disrupted the lives of young people in many ways, including education, mental health, and social interactions (6). In many countries, youth have been affected by measures implemented by decision-makers to control the spread of the virus, including lockdowns, school closures, and restrictions on social interactions (7). Trust in decision-makers and their response to the pandemic has been identified as a key factor influencing compliance with measures and public health outcomes (8).

The pandemic's impact on young people's mental health has been significant. A survey conducted by the United Nations and several studies found that the pandemic has increased stress, anxiety, and depression among young people (9–13). The pandemic has also affected the education of young people. School closures and the shift to online learning have resulted in significant disruptions to the education of millions of students worldwide (14). A report by UNESCO estimates that the pandemic has affected over 1.5 billion students and youth in more than 175 countries (14).

Risk Communication and Community Engagement (RCCE) and Infodemics are two new sciences that have been reintroduced and highlighted as essential fields that public health entities need to consider. RCCE became a critical component of public health emergency preparedness and response. RCCE involves the systematic and planned process of communicating health risks and engaging communities in response efforts to address a public health emergency. Effective RCCE helps increase awareness, build trust, and empower communities to protect themselves and their loved ones (15–17).

On the other hand, Infodemics is the spread of both accurate and inaccurate information during public health emergencies. With the rise of social media and instant communication, false information can cause confusion, panic, and harm. Infodemics can undermine public trust in health authorities and impede efforts to control a public health emergency. Addressing infodemics is crucial to ensure accurate information is disseminated and individuals can make informed decisions about their health. Collaboration between health authorities, media outlets, and other stakeholders can combat infodemics and promote public health during crises (18, 19).

The impact of the pandemic on young people has highlighted the need for policies and interventions to support their mental health, education, and overall well-being (11). Efforts to address the impact of the pandemic on young people have included providing mental health support services, developing online learning resources, and expanding youth employment and entrepreneurship programs (20).

Several studies have examined trust in decision-makers and its impact on public health outcomes during the COVID-19 pandemic (21–27). However, few studies have specifically focused on the trust of youth in decision-makers and their response to the pandemic (28). This study aims to address this gap in the literature by understanding the sources of information and confidence of youth and their trust in response to the pandemic and the main actors involved.

This analytical cross-sectional study utilized online platforms to conduct a survey. The survey focused on youth trust in the COVID-19 response and was disseminated via the Internet for a period of three months between May and August 2020. The survey was presented in the form of a web-based questionnaire that could be accessed through a clickable link on Facebook and WhatsApp social media platforms. Participation in the survey was completely voluntary and anonymous, and respondents could skip questions.

## Methodology

### Sample size and technique

For this study, non-random convenience sampling was utilized. The sample size was determined using EpiInfo software (29) and based on previous research that examined the effects of the COVID-19 pandemic on youth. Assuming 80% power, *p* < 0.05 level of significance, a 30% null hypothesis value, and an estimated proportion of 30%, the sample size was determined to be 266 respondents. Considering potential dropouts, estimated at a rate of 41.8% (30), the final sample size was calculated to be 377 respondents. Ultimately, the study aimed for responses from 412 respondents.

### Study tool and data collection technique

This web-based questionnaire was designed using Microsoft Forms and disseminated via a link shared on social media. The questionnaire consisted of several sections, including socio-demographic background, occupational data, questions to assess knowledge and attitudes regarding COVID-19, and questions to evaluate their confidence and trust in different actors involved in the COVID-19 pandemic. The questionnaire was adapted and modified from the World Health Organization's tool for behavioral insights on COVID-19 (29) and the Scottish Youth Parliament's survey of young people regarding their opinions on COVID-19 (30). A pilot testing phase was conducted to tailor and validate the questionnaire to the Egyptian context and confirm the items' and questions' clarity and comprehensibility. This survey was conducted over a period of three months between May and August 2020.

### Data management and analysis

Data coding and entry were conducted using Microsoft Forms and transferred to Excel before being exported to SPSS version 21 for statistical analysis. Descriptive statistics were utilized to summarise the data, including simple frequencies expressed as numbers and percentages for qualitative variables, mean and standard deviation for normally distributed quantitative variables, and median and quartiles for skewed quantitative variables. Comparisons between groups were evaluated using the appropriate statistical significance tests, and a *p*-value of ≤0.05 was deemed significant.

### Ethical consideration

The study received approval from the research ethics committee at Suez Canal University. Before participating in the online survey, informed consent was obtained from each participant following a comprehensive introduction to the study's objectives and the confidentiality measures that would be implemented to protect the collected data in accordance with the Helsinki Declaration (31). Participant's personal information was kept confidential throughout data collection, entry, and analysis. All information collected was anonymized.

## Results

### Demographics

The response rate was 98.5%, resulting in 406 responses out of 412, which were mainly single (91.1%) youth with a median age of 22. Males and females represented; 41.8% and 58.6%, respectively. Ninety-five percent received university or postgraduate education, of which 65.3 percent were in the medical field, and 82.3% of the total live in rural areas. Only about 28.6% were working individuals and considered themselves of moderate family income. Any chronic illnesses were denied by 375 (92.4%). The full demographic information can be found in Table 1.

**Table 1:** The demographic characteristics of the respondents.

Item		Number	Percentage
Agree to fill out the questionnaire	Yes	406	98.5%
	No	6	1.5%
Age (Median ± IQR)		(22 ± 20–25)	
Item		Number	Percentage (of all respondents, *N* = 406)
Gender	Male	168	41.4%
	Female	238	58.6%
Education degree	Preparatory or less	3	0.6%
	Secondary	19	4.7%
	University or postgraduate	384	94.6%
Type of Study	Medical	244	63.5%
	Non-medical	140	36.5%
Marital status	Married	35	8.6%
	Single	370	91.1%
	Divorced/widowed	1	0.2%
If married or divorced, has children?	Yes	30	78.9%
	No	8	21.1%
Residence	Urban	72	17.7%
	Rural	334	82.3%
Occupation	Works	116	28.6%
	Doesn't work	56	13.8%
	Student	234	57.6%
If it works, the field of occupation	Medical	60	51.7%
	Non-medical	56	48.3%
Economic status	Expenses exceed income	116	28.6%
	Expenses equal income	193	47.5%
	Expenses less than income	97	23.9%
Chronic illness	Yes	31	7.6%
	No	375	92.4%

### Background information

84% received information on self-prevention, and 93% were updated with the availability of treatment measures when the survey was conducted. 85% knew the maximum incubation period for the virus to be 14 days. Fever, cough, anosmia, ageusia, and diarrhea were known as COVID-19 symptoms by 99%, 93%, 80%, and 63%, respectively. The main intervention 92.1% of respondents adopted was to wash their hands regularly using soapy water or an alcohol-containing detergent.

### Source of information

**Figure 1:**
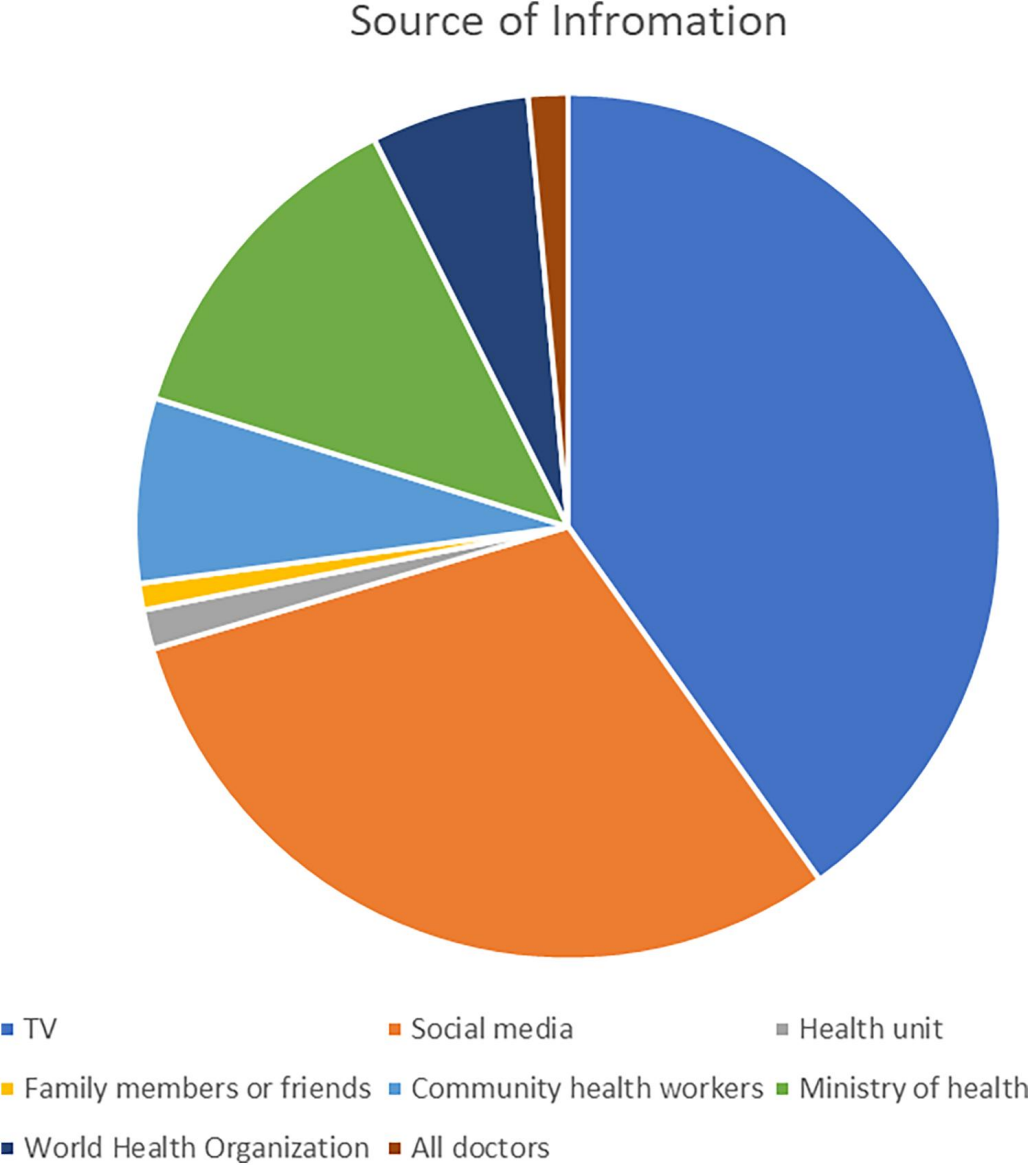
The sources of information.

We asked the participants about their sources of information and news about the pandemic. T.V. constituted 163 (40.1%), social media 123 (30.3%), Ministry of Health 52 (12.8%), Community health workers 28 (6.9%), and doctors 6 (1.5%) of the participants' sources of information. The full breakdown can be found in Figure 1. The total table can be found in Supplementary Annex 1.

Furthermore, when investigating the association between the different channels, a significant association was found between the source of information and the respondents' job. This was based on the 116 respondents working in medical or non-medical fields, with 60 and 56 responses, respectively. The breakdown can be found in Table 2.

**Table 2:** The association between the main channels of information and the job field of the respondents.

			What channels or sources have you heard about the new Coronavirus?									*P*-value
			T.V.	Social media	Health unit	Family members or friends	Community Health workers	Ministry of health	World Health Organization	All doctors	Total	
Job	Medical	Count	18	18	1	0	8	6	5	4	60	0.05
		% within Job	30.00%	30.00%	1.70%	0.00%	13.30%	10.00%	8.30%	6.70%	100.00%	
	Non-medical	Count	32	10	1	0	4	7	1	1	56	
		% within Job	57.10%	17.90%	1.80%	0.00%	7.10%	12.50%	1.80%	1.80%	100.00%	
Total		Count	50	28	2	0	12	13	6	5	116	
		% within Job	43.10%	24.10%	1.70%	0.00%	10.30%	11.20%	5.20%	4.30%	100.00%	

### Confidence in the key actors

**Figure 2:**
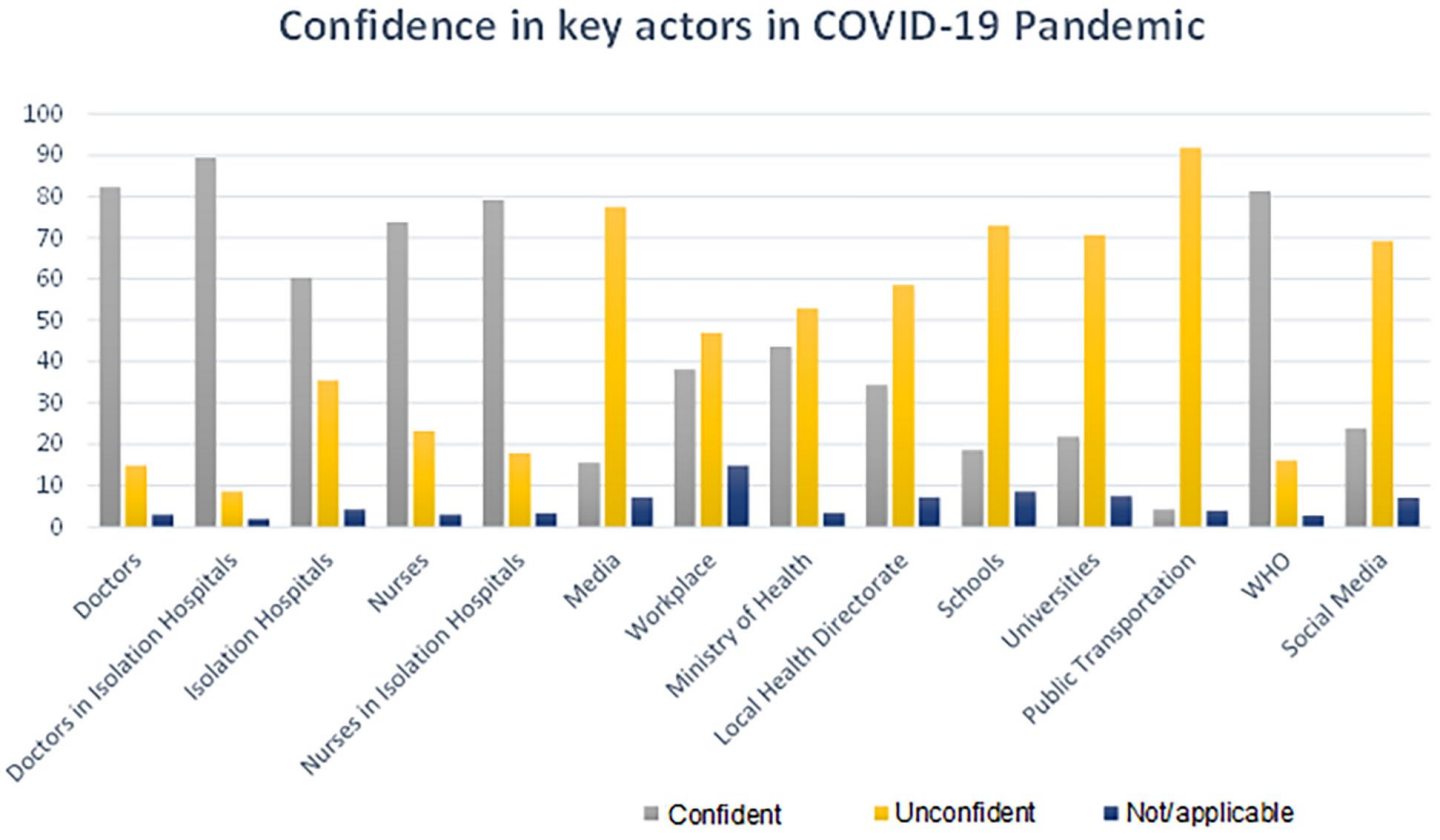
The confidence in key actors in the COVID-19 pandemic.

The respondents were asked about their confidence in key actors in the COVID-19 pandemic, particularly those sharing information and included in the decision-making process and implementing different public health and social measures (PHSM). 315 (77.6%) of the respondents were concerned with the decisions taken by key actors in the pandemic situation, while 61 (15%) were neutral, and 22 (5.4%) were not concerned. 363 (89.4%) of the respondents were most confident in doctors working at isolation hospitals, While 334 (82.3%) showed confidence in doctors mainly. On the other hand, 17 (4.2%) of the respondents were least confident in the public transportation system and 63 (15.5%) in conventional media outlets. The full breakdown is shown in Figure 2 and Supplementary Annex 2.

## Discussion

A high response rate of 98.5% of 406 participants reflected a friendly and practical questionnaire layout adding more credibility to the answers submitted. The fact that most respondents were students of medical background limits such results to such a population and did not necessarily reflect awareness and knowledge in the community. However, these results represent the views and opinions of young people who actively represent a major future working force in Egypt (32). Preventive measures, as well as disease symptoms, were widely recognized by a good 90.2% of the respondents indicating significant outreach programs in place. Washing hands with soapy water or alcohol-based products were successfully adopted by 92.1%. Other measures such as covering mouth and nose when coughing or sneezing, wearing a mask, avoiding close contact with symptomatic individuals, and home isolation were not as seriously considered as washing hands, Indicating to some extent the success of hand washing campaigns by the Egyptian Ministry of Health that started with the pandemic (33). Financial ability to buy masks, personal protective equipment (PPE) availability in stores or hospitals, or a perceived socially-drastic measure such as home isolation could be factors to consider for such a response, which contradicts data produced by other studies targeting Egyptian healthcare workers (34).

A big portion of respondents (77.6%) expressed their concern about the pandemic response. This indicated a decreased perceived value to the updates and announcements by key actors in the pandemic and low confidence in the health system leading the response, which is supported by other literature on Egyptian medical students in similar fields (35). Thus, building confidence in the response system to pandemics is vital and has been extensively discussed as a priority in several publications (36, 37).

Social media and T.V. were reported to be the main sources of information despite their lack of confidence in them. Figure 2 shows that most participants were confident in medical personnel directly in contact with patients -doctors and nurses-. It raises concern whether the participants recognize the bigger role, the bigger healthcare institutions play in controlling such an emergency state during the pandemic. This is supported by other literature, especially with the conspiracy theories around the COVID-19 pandemic getting more publicity (38).

Regarding confidence in the source of information regarding COVID-19, doctors, nurses, and other healthcare professionals were at the center of the network of trust, which is reasonable and concurrent with results from other studies (39). However, in contrast to data in other countries, there was low confidence in the media and official reports (40). This, in particular, considering the confidence in WHO reports, supports claims of the presence of discrepancies between what was reported by the WHO and that of the media during the early phases of the pandemic (41–43).

Similar theories were reported article by Tuite et al., who estimated the number of cases in Egypt to be much larger than reported (44). However, Hassany et al. later argued that the validity of the used model for estimation had many scientific fallacies and couldn't be generalized to the Egyptian context (45). While these scientific arguments were very productive, the media was only concerned with reporting the first, leaving the general population overwhelmed and questioning the official reports (41–43).

Nevertheless, though most respondents were most confident in doctors working at isolation hospitals and were least confident in the conventional media outlets when asking about the source of information regarding COVID-19, 70.4% of the respondents reported media and T.V. as their primary sources of information. Only 7.4% reported doctors and WHO platforms as their primary sources. This is unsurprising as similar results were reported in previous literature (46, 47).

This supports all the literature on the role of media and the new science of Infodemics. WHO defines it as “too much information, including false or misleading information in digital and physical environments during a disease outbreak” (18). During COVID-19 and the rising influence of social media being a primary source of information, infodemics, including rumors, conspiracy theories, and stigma, have made it difficult to control, prevent and manage the disease in addition to their role in public adherence to PHSM and vaccine hesitancy (19, 48, 49).

Given the above mentioned and considering the implications of infodemics on comprehensive health literacy (50), there should be new strategies to improve health information literacy within the general population. One important model to consider was the one applied in Singapore in 2006 (51).

This study has several limitations. Since the conduction of this study, there have been advancements in our comprehension of the virus and efforts to reduce its transmission. Consequently, subsequent survey waves should encompass new topics to capture up-to-date information on behaviors and other relevant issues. This study was conducted on a sample of young people in Egypt through an online platform, which was challenging during the initial stages of the COVID-19 pandemic with the extensively applied measures. Online surveys have many limitations pertaining to their generalizability and exclusively reaching those with an internet connection, which may bias the results (52, 53). However, they still have advantages, especially in such unprecedented pandemic times (54, 55). Therefore, our findings may not be universally applicable, particularly to adults. Additionally, as stated in the results, a small proportion of individuals declined to participate in the study.

Descriptive research is useful in identifying the extent of a problem. Still, it is essential to have a sound theoretical basis for making sense of the findings and formulating effective measures to prevent and treat them. In the context of events of public health concerns, and ultimately public health generally, it is necessary to maintain trust between authorities and other information-sharing channels with the community through effective communication strategies. RCCE has been reintroduced in the context of COVID-19 as an essential public health function of effective health systems (17). This underscores the importance of successful communication methods to establish confidence, encourage compliance with public health measures, and lessen the impact of false information and rumors about COVID-19. We hence recommend further interventional mapping studies adopting new techniques to enhance access to credible and evidence-based health information within the public. We also recommend capitalizing on the technological and scientific advancements and building future RCCE strategies based on our understanding and meaningful inclusion of the target populations, deploying different sciences such as behavioral surveillance (56, 57) and digital health communications and tools (58, 59).

In conclusion, no doubt COVID-19 has influenced how people seek and perceive health information. Though the health implications were disastrous, it is time to benefit from that experience in reframing how health information is delivered to the public. Therefore, adopting effective RCCE strategies is critical to building trust in the normal time. COVID-19 was an unprecedented crisis, despite several warnings by many experts (60), so it's essential to learn from our pitfalls and be better prepared for future outbreaks. Hence, action should be taken by public health authorities and governments to improve comprehensive health information literacy and develop information technology strategies that promote evidence-based information and combat widespread misinformation.

## Data Availability

The original contributions presented in the study are included in the article/Supplementary Material, further inquiries can be directed to the corresponding author.
