# Supplementary material for: Youth perspectives on information and trust during COVID-19: evidence from Egypt
Source: Front Digit Health. 2025 Sep 8;7:1563203. doi: 10.3389/fdgth.2025.1563203 (PMC12450864; doi:10.3389/fdgth.2025.1563203)
Supplement: Supplementary file 1 [file Datasheet1.docx]

**Supplementary tables: "Youth Perspectives on Information and Trust During COVID-19: Evidence from Egypt"**

**Annex 1**

|  | | What channels or sources have you heard about the new Corona virus? | | | | | | | |  |
| --- | --- | --- | --- | --- | --- | --- | --- | --- | --- | --- |
|  |  | TV | Social media | Health unit | Family members or friends | Community health workers | Ministry of health | World Health Organization | All doctors | Total |
| Total | Count | 163 | 123 | 6 | 4 | 28 | 52 | 24 | 6 | 406 |
|  | Percentage | 40.10% | 30.30% | 1.50% | 1.00% | 6.90% | 12.80% | 5.90% | 1.50% | 100.00% |

**Annex 2**

| Actor | | Very Confident | Confident | Unconfident | Very Unconfident | Not Applicable | Total |
| --- | --- | --- | --- | --- | --- | --- | --- |
| Isolation Hospitals | Frequency | 86 | 159 | 111 | 33 | 17 | 406 |
|  | Percent | 21.2 | 39.2 | 27.3 | 8.1 | 4.2 | 100 |
| Doctors | Frequency | 138 | 196 | 43 | 17 | 12 | 406 |
|  | Percent | 34 | 48.3 | 10.6 | 4.2 | 3 | 100 |
| Doctors in Isolation Hospitals | Frequency | 180 | 183 | 22 | 13 | 8 | 406 |
|  | Percent | 44.3 | 45.1 | 5.4 | 3.2 | 2 | 100 |
| Nurses | Frequency | 96 | 204 | 71 | 23 | 12 | 406 |
|  | Percent | 23.6 | 50.2 | 17.5 | 5.7 | 3 | 100 |
| Nurses at Isolation Hospitals | Frequency | 120 | 201 | 51 | 21 | 13 | 406 |
|  | Percent | 29.6 | 49.5 | 12.6 | 5.2 | 3.2 | 100 |
| World Health Organization | Frequency | 138 | 192 | 48 | 17 | 11 | 406 |
|  | Percent | 34 | 47.3 | 11.8 | 4.2 | 2.7 | 100 |
| Ministry of Health | Frequency | 59 | 118 | 119 | 96 | 14 | 406 |
|  | Percent | 14.5 | 29.1 | 29.3 | 23.6 | 3.4 | 100 |
| Local Health Directorate | Frequency | 25 | 114 | 162 | 76 | 29 | 406 |
|  | Percent | 6.2 | 28.1 | 39.9 | 18.7 | 7.1 | 100 |
| Media | Frequency | 11 | 52 | 168 | 146 | 29 | 406 |
|  | Percent | 2.7 | 12.8 | 41.4 | 36 | 7.1 | 100 |
| Social Media | Frequency | 13 | 84 | 193 | 88 | 28 | 406 |
|  | Percent | 3.2 | 20.7 | 47.5 | 21.7 | 6.9 | 100 |
| Schools | Frequency | 10 | 65 | 202 | 94 | 35 | 406 |
|  | Percent | 2.5 | 16 | 49.8 | 23.2 | 8.6 | 100 |
| Universities | Frequency | 16 | 73 | 184 | 103 | 30 | 406 |
|  | Percent | 3.9 | 18 | 45.3 | 25.4 | 7.4 | 100 |
| Public Transportation | Frequency | 7 | 10 | 150 | 223 | 16 | 406 |
|  | Percent | 1.7 | 2.5 | 36.9 | 54.9 | 3.9 | 100 |
| Workplace | Frequency | 30 | 125 | 114 | 77 | 60 | 406 |
|  | Percent | 7.4 | 30.8 | 28.1 | 19 | 14.8 | 100 |
